# Establishment and characterization of transformed goat primary cells by expression of simian virus 40 large T antigen for orf virus propagations

**DOI:** 10.1371/journal.pone.0226105

**Published:** 2019-12-05

**Authors:** Yumiko Yamada, Guan-Ru Liao, Ching-Yu Tseng, Yeu-Yang Tseng, Wei-Li Hsu

**Affiliations:** 1 Graduate Institute of Microbiology and Public Health, National Chung Hsing University, Taichung, Taiwan; 2 Department of Immunology and Infectious Diseases, John Curtin School of Medical Research, Australian National University, Canberra, Australia; Kansas State University, UNITED STATES

## Abstract

Due to the limited host range of orf virus (ORFV), primary cells derived from its natural hosts, such as goats and sheep, are recommended for isolation and propagation of wild type ORFV. This situation limits the option for the study of virus-host interaction during ORFV infection since primary cells only support a few numbers of passages. SV40 T antigen is a viral oncoprotein that can abrogate replicative senescence, leading to an extended life span of cells. In this study, the transformation of two goat primary cells, fibroblast (FB) and testis (GT) cells, were achieved by stably expressing SV40 T antigen using the lentiviral technique. The presence of the gene encoding SV40 T antigen was validated by polymerase chain reaction (PCR) and western blot analyses. As evidenced by immunofluorescent microscopy, the two types of cells expressing SV40 T antigen (namely, FBT and GTT) were purified to homogeneity. Moreover, faster growth kinetics and a lower serum dependency were noticed in FBT and GTT, as compared with their counterpart parental cells. FBT and GTT remain permissive and can form plaque of ORFV, despite with different profiles; generally speaking, with SV40 T expression, ORFV forms plaques with smaller size and distinct margin. Most importantly, the prolonged life span of goat FBT and GTT serves as an ideal cell culture resource for ORFV isolation from the field, studies of ORFV pathogenesis and efficient vaccine development.

## Introduction

Orf virus (ORFV) is a member of *Parapoxvirus* genus, *Poxviridae* family and it consists of a linear double-stranded genome approximately 138 kb in length [1]. ORFV infection causes contagious ecthyma, which mainly affects sheep and goats [2] as well as other ruminants such as serows [3], tahr, steenboks, and chamois [4]. Symptoms in infected animals include proliferative lesions in the skin of the nostrils, lips, oral mucosa, gums and tongue [5]. ORFV infection can reach up to 10% mortality in lambs and 93% mortality in kids [6]. This high mortality case found in young animals was due to its inability to suck and intake nutrition properly [7]. Besides, bacterial and fungal secondary infections are commonly noticed after primary ORFV infection, resulting in another wave of economic loss [8]. ORFV is considered a zoonotic etiologic pathogen particularly in veterinarian, shepherds, and butchers [9]. Infection in humans usually manifested as a single papule on fingers, hands or other body parts, which is accompanied by lymphadenopathy, fever or malaise [5]. In most of the situations, the disease is self-limiting and can heal without treatment in three to six weeks. However, in immunocompromised patients, large lesions could be developed [10].

Isolation and propagation of biologically active viruses are essentials for virology researches in various fields, such as vaccine designs, antiviral drug developments [11] and novel strategies for cancer treatment by viral vectors [12–18]. Poxvirus can infect both permissive and restrictive cells and downstream intracellular signaling plays a determinant role in the host tropism of the virus [19]. Although specific host-cell receptors have not been identified, poxvirus can potentially bind and enter an extensive range of mammalian cell lines [20]. However, unlike the classic poxvirus, infection of ORFV causes peripheral lesions and highly adapts to skin exclusively [21]. Based on previous literature, isolation of ORFV can be efficient only in specific primary cells, including cells produced from lamb testis and kidney, fetal lamb muscle and turbinate and fetal bovine muscle and lung [22–26]. However, organs for the establishment of primary cells are normally obtained from sacrificed animals and most primary cells can only be propagated for limited passages due to growth arrest and irreversible senescence.

The limited life span of primary cells can be overcome by the generation of immortalized cells using several methods. By utilization of human telomerase reverse transcriptase (hTERT), cells with an exogenous hTERT gene acquire the ability to proliferate continuously by telomerase activation [27]. This technique has been utilized to establish cell lines derived from muscular epithelial cells [28], trophoblast cells [27, 29], microglia cells [30], fibroblast cells [31], and mammary epithelial cells [32]. Furthermore, immortalization can be achieved by targeting *K-ras*, a gene known to regulate cell growth, proliferation, and differentiation. K-ras mutation plays a significant role in immortalization by suppressing the hydrolysis of guanosine triphosphate, leading to the upregulation of downstream signaling activity and then uncontrolled proliferation and resistance to apoptosis [33].

In addition to these two methods, another approach to generate a transformed cell line is by introducing the *large T* gene of simian virus 40 (SV40), belonging to a genus of polyomaviruses. This virus can infect a wide range of cells, including those derived from rodents [34], sheep [35–38], goats [37, 39] and humans [40–43]. SV40 T antigen is the major viral factor contributing to the SV40-mediated cell transformation. SV40 T antigen exerts this effect by binding and thereby, inactivating a variety of cellular proteins, which are responsible for driving the cells into S-phase and for promoting cellular division, such as retinoblastoma (Rb) and p53 tumor suppressor as well as inhibiting protein phosphate 2 (PPP2) [44]. Also, the role of the T antigen on the Rb-family is to release cells from growth arrest [45]. The hallmark of phenotypic changes in cell transformation includes growth to high saturation density, focus formation, growth in soft agar, serum-independence, morphological changes, elevated glucose uptake, and plasminogen activator production [44]. Furthermore, SV40-transformed cells can induce tumor formation when inoculated into immunocompromised mice and other tested animals [46].

In our study, the transformation of goat primary cells, fibroblast (FB) and testis (GT) cells, were carried out by transduction of lentivirus particles expressing the *SV40 T antigen* gene. These cells were characterized according to their morphology, fetal bovine serum (FBS) dependency, susceptibility to ORFV, plaque formation ability and yields of virus progeny. The results of this study suggested that SV40-transformed goat cells (FBT and GTT cells) will be beneficial for investigating the pathogenesis of ORFV and developing vaccines without considering the limited life span of primary cells.

## Materials and methods

### Cell maintenance

The two primary goat cells used in this study were prepared by Lin et al. (2015) [11]. In brief, one two-week old lamb (Nubian breed) was sacrificed followed by the removal of its testis and ears. The protocols used in this procedure were approved by the Committee on the Ethics of Animal Experiments of National Chung Hsing University (approval numbers: 101–40). The derived testis cells and fibroblasts from ears were cultured in 1× RPMI 1640 medium with 10% fetal bovine serum (FBS). When cells grew to full confluency, cells were trypsinized and subcultured in 1:1 or 1:2 ratios.

Human embryonic kidney cell 293T (HEK293T), African green monkey cells (Vero cells), human lung carcinoma cell line A549, goat FB cells and GT cells, and SV40-transformed cells (FBT and GTT) were propagated in Dulbecco’s modified Eagle medium (DMEM; Gibco BRL, Life Technologies Corporation, Carlsbad, CA, USA) supplemented with 10% FBS (Hyclone, Logan, UT, USA) and 1% penicillin-streptomycin (Gibco BRL). Madin-Darby Canine Kidney (MDCK) and was grown in DMEM supplemented with 5% FBS and 1% penicillin-streptomycin. All cells were incubated at 37°C with 5% CO_2_.

### Production of lentivirus and transduction

Lentivirus was packaged in HEK293T cells. Large T antigen transfer vector was purchased from Addgene (# 18922). For negative control, we also generated the lentiviral vector without insertion of the gene of large T antigen (namely EV) by digestion of the original large T antigen transfer plasmid with restriction enzyme, *EcoR*I, followed by self-ligation. In brief, second-generation lentiviral packaging plasmid (psPAX2), envelop plasmid (pVSVG) and the transfer plasmid (with or without insertion of the gene encoding large T antigen) were co-transfected into HEK293T at the ratio of 2:1:4 by lipofectamine 3000 (Invitrogen). The supernatant containing packaged lentivirus in the transfected cells was harvested at 24, 48 and 74 hours after transfection, followed by centrifugation at 12,000 g to concentrate the virus. The concentrated virus was used to transduce FB and GT cells in the presence of 8 μg/ml of polybrene (Santa Cruz). At 24 hours post-transduction, FB and GT cells were selected by using puromycin at concentration of 3 μg/ml and 5 μg/ml, respectively, and incubated at 37°C for another 24 hours, followed by limiting dilution and single-cell isolation.

### Isolation of single-cell colonies expressing SV40 T antigen

Under puromycin selection, the survived transduced FB and GT cells were harvested in DMEM containing 10% FBS and 1.1% methylcellulose and various cell counts, e.g. 20, 50, or 200 cells were seeded into individual 35mm petri-dish for colony formation. Several single colonies were randomly picked and transferred to a 24-well plate for further amplification, followed by genotyping and phenotyping.

### Growth analysis

Both goat primary cells and SV40 T-transformed cells were seeded in a 48-well plate (1.0x10^4^/well) and the growth of these cells was determined by counting the total viable cells at 24 and 48 hours after the seeding. The cells were maintained at different FBS levels (2.5%, 5%, and 10%). Cell count was carried out in triplicates at each time point and the average of total cell counts was used to plot the growth curve.

### Virus and infection

OV20.0-GFP, a recombinant ORFV expressing enhanced green fluorescent protein (eGFP), was used to evaluate the susceptibility of different cell lines used in this study [47]. For infection, cells at 80% confluency were washed with phosphate-buffered saline (PBS) and then infected at the indicated multiplicity of infection (MOI) in DMEM without FBS. After one hour of incubation, the infection medium was removed and replaced with fresh DMEM containing 2.5% FBS, and plates were incubated at 37°C with 5% CO_2_.

### Plaque assay

GTT cells were seeded one night before infection and then were infected with 10-fold serially diluted viruses. One-hour post adsorption, the infectious medium was replaced with 1.1% methylcellulose in DMEM with 2.5% FBS. Infection was monitored daily until when the cytopathic effect (CPE) was visible (~6 days). The cells were then fixed by 10% formaldehyde and stained with crystal violet.

### Western blot analysis

Proteins were resolved by 12% sodium dodecyl sulfate-polyacrylamide gel electrophoresis and then electrophoretically transferred to a polyvinylidene difluoride membrane, followed by blocking using 5% skimmed milk for one hour. Primary immunoblotting with diluted antibodies, such as anti-SV40 T Antigen (1:5000, GeneTex134378), anti-F1L (1:2000, homemade), anti-alpha Tubulin (Novus NB100-690) and anti-GAPDH (1:5000, GeneTex627408), was done overnight at 4°C. Goat anti-mouse or rabbit IgG-conjugated with HRP antibodies were used as the secondary antibodies and incubated with the membrane for one hour at room temperature. After washing the membrane with 0.05% Tween 20 in PBS, the signal was revealed by an enzyme-linked chemiluminescence assay (SuperSignal, Thermo Scientific). The image was captured using ImageQuant LAS 4000 (GE Healthcare, Uppsala, Sweden) and the intensity of each band was determined by densitometry (ImageJ, NIH).

### Confirmation of genotypes by PCR

Cellular DNA was extracted using the genomic DNA kit (AllPure Genomic DNA Extraction Kit, AllBio Life), according to the manufacturer’s instructions. The presence of the SV40 T antigen gene and puromycin resistant gene (PuroR) in transduced cells were validated by PCR using the primers, SV40 T antigen-F: 5′-GATCTGCCTGAGGTGTTACTTG-3′ and SV40 T antigen-R: 5′-GGATGGCATCACTAGTCATGAC-3′; PuroR-F: 5′-ATGACCGAGTACAAGCCCAC-3′ and PuroR-R: 5’-TCAGGCACCGGGCTTGCG-3’. PCR was conducted by an initial denaturation step of 95°C for 5 min, followed by 30 cycles of 95°C for 10 sec, 55°C for 30 sec, 72°C for 30 sec, and a final extension of 72°C for 10 min. After amplification, 10 μL of PCR products were analyzed by 1% agarose gel and visualized under UV light.

### Immunofluorescence assay

Cells at 50 to 60% confluency were fixed with 1% formalin for 10 minutes followed by permeabilization (1% NP40 in PBS) for 10 minutes. Incubation of primary antibody, anti-SV40-T antigen (1:1000, GeneTex134378), was carried out at room temperature for one hour. Anti-Alexa Fluor 488 was used as a secondary antibody and incubated for one hour in the dark. Several washes of PBS containing 1% FBS were done between each step at room temperature. Fluorescence was observed by fluorescence microscopy.

### Statistical analysis

The data were expressed as the mean ± standard deviation. The two-way ANOVA was used in serum dependency to determine the growth kinetics of goat primary cells and SV40-transformed cells. Moreover, student T-test was performed to evaluate the susceptibility of the primary cells in compared with its respective SV40-transformed cells against ORFV. These two statistical tests were performed by GraphPad Prism version 6.00 (GraphPad Software). A p-value < 0.05 was considered significant and is represented by the asterisk sign, e.g. *, **, *** indicated p-value < 0.05, 0.01, 0.001, respectively, on the figures.

## Results

### Susceptibility of different cell lines to ORFV

**Fig 1:**
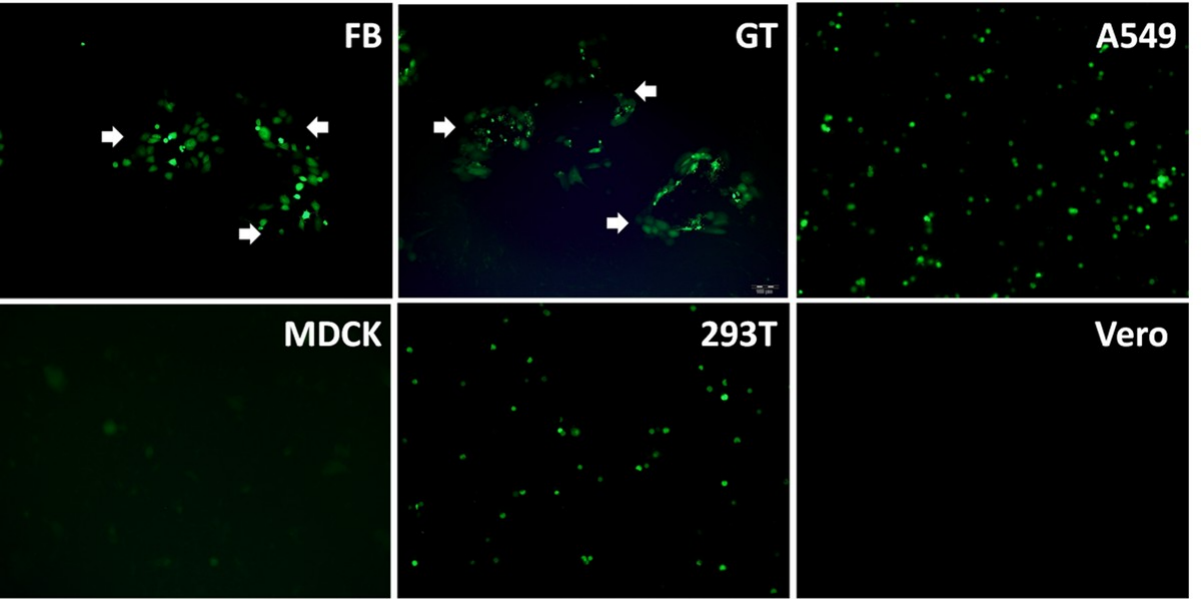
Susceptibility of different cell lines against ORFV. Two types of goat primary cells (FB and GT cells), human cells (A549, HEK293T), MDCK cells, and Vero cells were infected with OV20.0-GFP ORFV at 1 MOI. After 6 days post-infection (dpi), green fluorescence was observed under fluorescence microscopy.

To evaluate the ability of ORFV to infect different hosts, selected cells originated from different species were infected with OV20.0-GFP ORFV at 1 MOI. The ORFV was able to infect five out of six cell lines (i.e. FB cells, GT cells, A549 cells, HEK293T cells, and MDCK cells) tested as shown by the green fluorescence at 24 hours post-infection (hpi) (Fig 1). Noticeably, only FB and GT cells formed plaques as indicated by white arrows in Fig 1 at 6 days post-infection (dpi). Although Vero cells were used to propagate ORFV strain D1701 in the previous report [48], Vero cells were not permissive to OV20.0-GFP ORFV in the present study as evidenced with no green fluorescence signal observed at 6 dpi. Collectively, ORFV infection is in a cell-type dependent manner.

### Generation and validation of SV40-transformed cell lines that harbor T antigen

**Fig 2:**
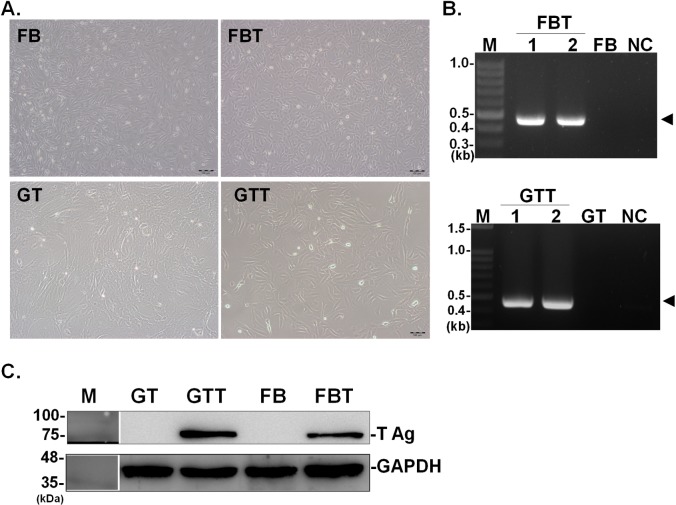
Morphology, phenotypic and genotypic analysis of the SV40-transformed cells. (A) Cell morphology was observed under bright-field microscopy. (B) PCR and (C) western blot analysis were further used to confirm the presence of the gene and expression of SV40 T antigen in goat fibroblast and testis cells. The same filter was incubated with the antibody for large T and GAPDH, as an internal control. FB and GT cells were served as respective parental cells for the assays.

**Fig 3:**
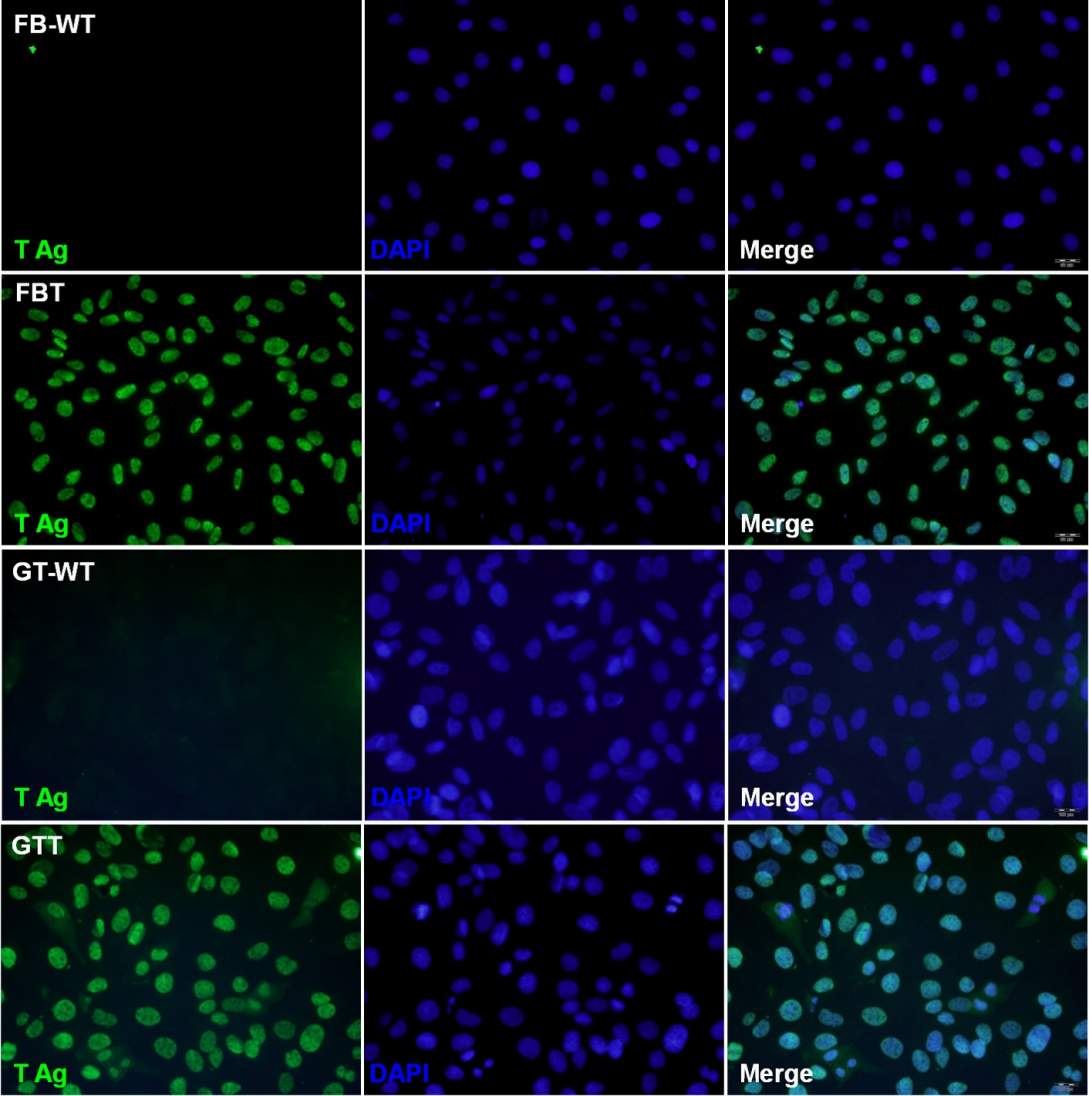
Immunofluorescence staining of goat fibroblast and testis cells. Parental FB (FB-WT), GT (GT-WT), and the SV40 T transformed FBT, GTT cells were stained with the large T-specific antibody. Expression of SV40 T (labeled as T Ag) was shown as green fluorescence, and DAPI counterstaining indicates the cell nuclei.

Due to the limited life span of primary cells, we aimed to establish immortalized goat cells by introducing the SV40 T antigen gene delivered by lentivirus. Single-cell colonies were isolated and grown under puromycin selection for more than 10 passages. Under bright-field microscopy (Fig 2A), the morphologies of FBT and GTT cells were indistinguishable from their respective primary cells. Next, the integration and expression of the SV40 T antigen gene were analyzed. PCR results demonstrated the presence of the SV40 T gene in both FBT and GTT cells (Fig 2B) suggesting the successful integration of SV40 T antigen. Consistently, expression of SV40 T antigen, with an expected size of 80 kDa, was detected at a comparatively high level in both FBT and GTT cells, whereas it was not detected in both FB and GT cells (Fig 2C). Moreover, to confirm whether FBT and GTT cells were purified to homogeneity, an immunofluorescent assay was performed. As shown in Fig 3, green fluorescence was detected in all FBT and GTT cells, indicating the purity of these cells. Taken together, the data showed that FBT and GTT cells had been successfully transduced with SV40 T antigen and high homogeneity was achieved by using puromycin treatment and single-cell colony growth.

### Serum dependence of goat primary cells and the SV40-transformed cells

**Fig 4:**
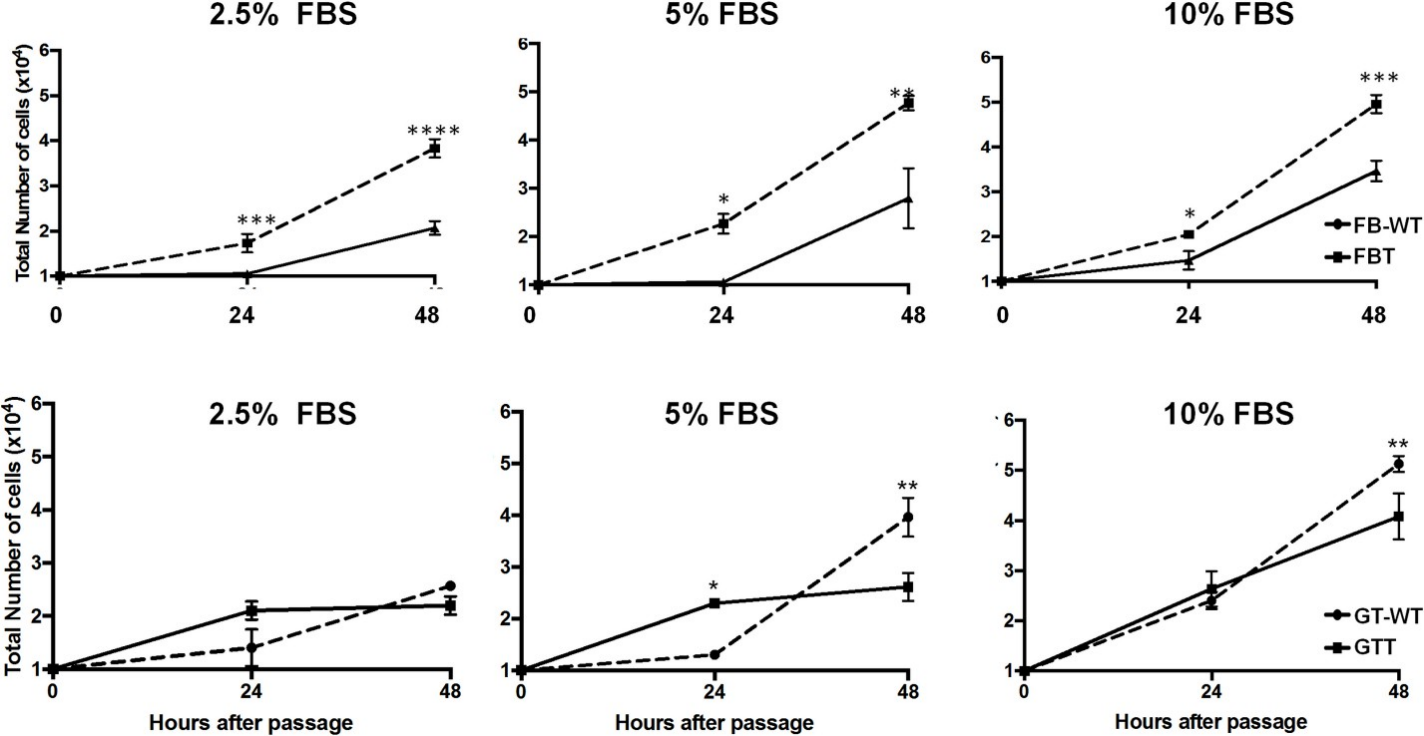
Growth curve of FB-WT, GT-WT, FBT, and GTT cell lines in different FBS levels. Two sets of cells, FB and FBT (upper panel), as well as GT and GTT (lower panel), were seeded at the same density (1x10^4^ cells/well) in a 48-well plate and were cultivated in DMEM supplemented with 10%, 5% or 2.5% of FBS. Total cell counts were determined at 24 and 48 hours after seeded. The experiment was conducted in three repeats and the average cell counts were plotted. Two-way ANOVA was used to determine the growth kinetics of goat primary cells and SV40-transformed cells in different FBS level at different time points. A p-value < 0.05 was considered significant and is represented by the asterisk sign, e.g. *, **, *** indicated p-value < 0.05, 0.01, 0.001, respectively.

Due to the constitutive expression of SV40 T antigen in FBT and GTT cells, we further investigated whether SV40 T antigen influences the growth rate in a serum-dependent manner in these transformed cells. In general, higher concentrations of FBS in DMEM medium led to a faster growth rate in both types of goat primary cells. In particular with 5 or 10% FBS supplement, FBT and GTT cells amplified to a significantly higher degree than their respective parental cells (Fig 4). Moreover, FBT and GTT cells had a faster growth rate than FB and GT cells between 24 to 48 hours after seeded, and it reached to full confluency in a shorter time, even in 2.5% of FBS growth condition (Fig 4). To further confirm the cell growth were indeed due to the expression of SV40 T antigen, but not the effect of lentiviral vector integration, we evaluated the cell growth kinetics between parental cells and its respective cells transduced by lentivirus bearing empty vector (namely FB-EV and GT-EV). The presence of lenti-EV and morphology were initially monitored by PCR and microscopy (panel A and B in S1 Fig). Unlike in the cells with SV40 T antigen, the growth rate of FB-EV and GT-EV cells was not increased as compared with their corresponding parental cells (S1 Fig). Hence, the faster growth kinetics of SV40-transformed cells were due to the successful integration of SV40 T antigen and not due to the lentiviral function.

### Susceptibility of SV40-transformed cells to ORFV infection

**Fig 5:**
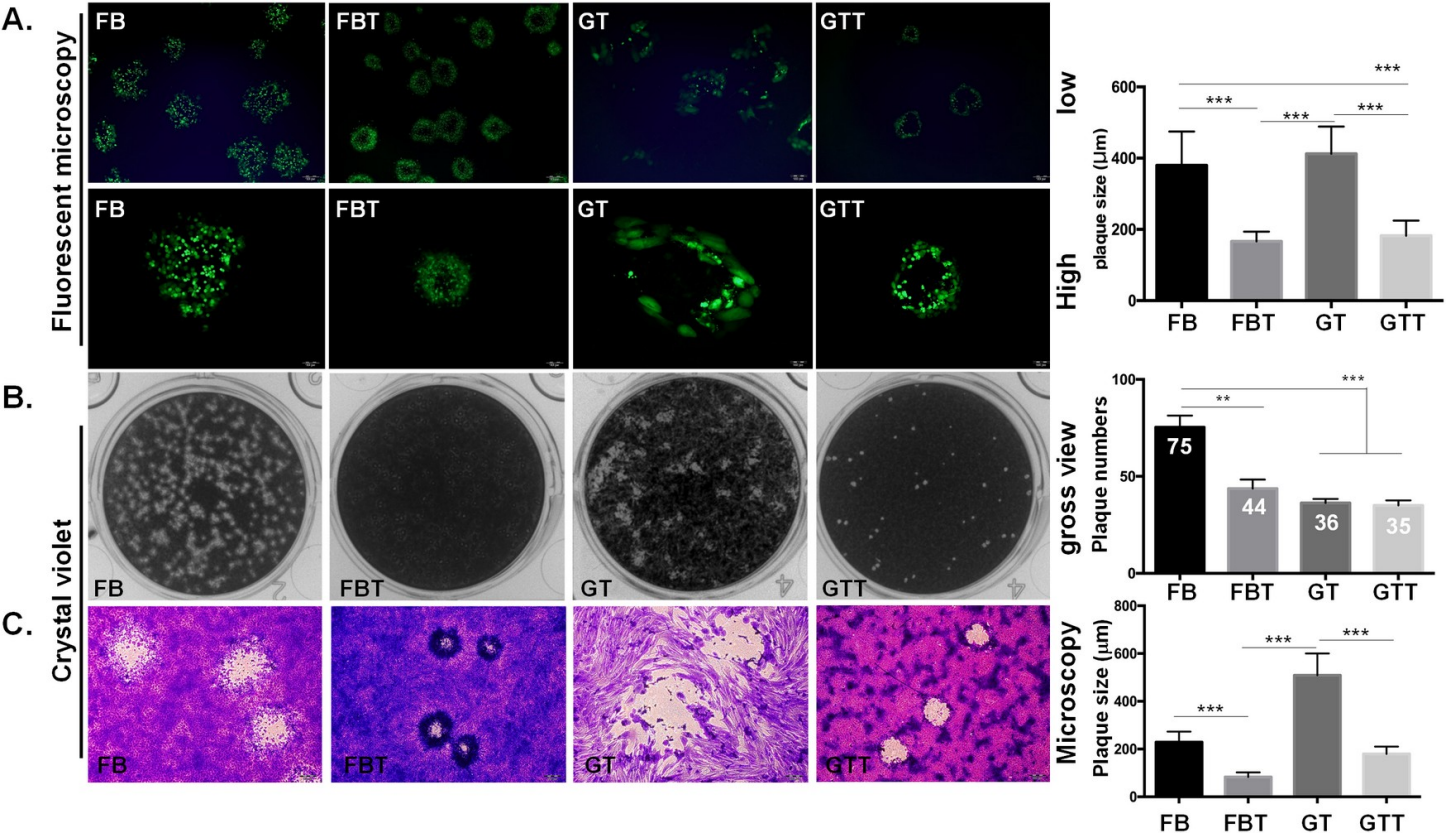
Plaque formation ability of ORFV in four different cell types. The plaque formation ability was determined based on plaque assay. Cells were infected with OV20.0-GFP at 100 PFU/well and grown in an infectious medium containing 1.1% methylcellulose. Plaques were monitored either by fluorescence microscopy at both high and low magnifications (A), or stained with crystal violet at 6 dpi (B and C). The number of plaques was counted by the gross view (B, right panel), and the plaque size was measured under fluorescence microscopy (A, right panel) and bright field microscopy (C, right panel). The student T-test was performed to evaluate the plaque formation of the primary cells in comparison with its respective SV40-transformed cells against ORFV. A p-value < 0.05 was considered significant and is represented by the asterisk sign, e.g. *, **, *** indicated p-value < 0.05, 0.01, 0.001, respectively.

An ideal host for the virus infection or as a cell model for virology study should support efficient virus propagation, the appearance of CPE and ultimately the emergence of plaques. ORFV can infect several cell lines, but it only forms distinct plaques in specific cell types, i.e. goat primary cells (Fig 1). Hence, we firstly evaluated the plaque formation ability of ORFV in FBT and GTT cells in comparison with their respective primary cells. By infection with 100 plaque formation unit (PFU) per well of OV20.0-GFP ORFV, fluorescence microscopy was used to monitor the plaque formation ability during the infection; moreover, crystal violet staining was utilized to visualize the yield plaques by naked eyes or via the bright filed microscopy. As shown in Fig 5, CPE was observed in the four cell cultures, although the size and morphology of ORFV plaques were distinct among these cells. In general, plaques were significantly larger in goat primary cells than in SV40 T expressing cells. By fluorescence microscopy, it revealed ORFV infection developed circle plaques with cell debris in the center in FB cells, while GT cells produced large and irregular shape of plaques. On the other hand, circle plaques with hollow in centers were observed in FBT cells as well as in GTT cells. Noticeably, the diameter of plaques was significantly larger in GT cells as compared with FBT and GTT cells (Fig 5A). At 6 dpi, plates were stained by crystal violet and observed under the naked eye (Fig 5B), or bright-field microscopy (Fig 5C). When the PFU was estimated by a gross view, FB cells yielded the highest number of plaques with a mean number of 75 PFU, which is approximately double of those in the other three cell types analyzed (Fig 5B, right panel). Consistent with the observation by fluorescent microscopy, GT cells yielded plaques with size significantly larger than the rest of the cell types (Fig 5C). However, smaller plaques were observed in SV40-transformed cells, in particular with FBT cells, as compared with their respective primary cells; indicating SV40 T antigen expression slightly influences the plaque formation during ORFV infection.

**Fig 6:**
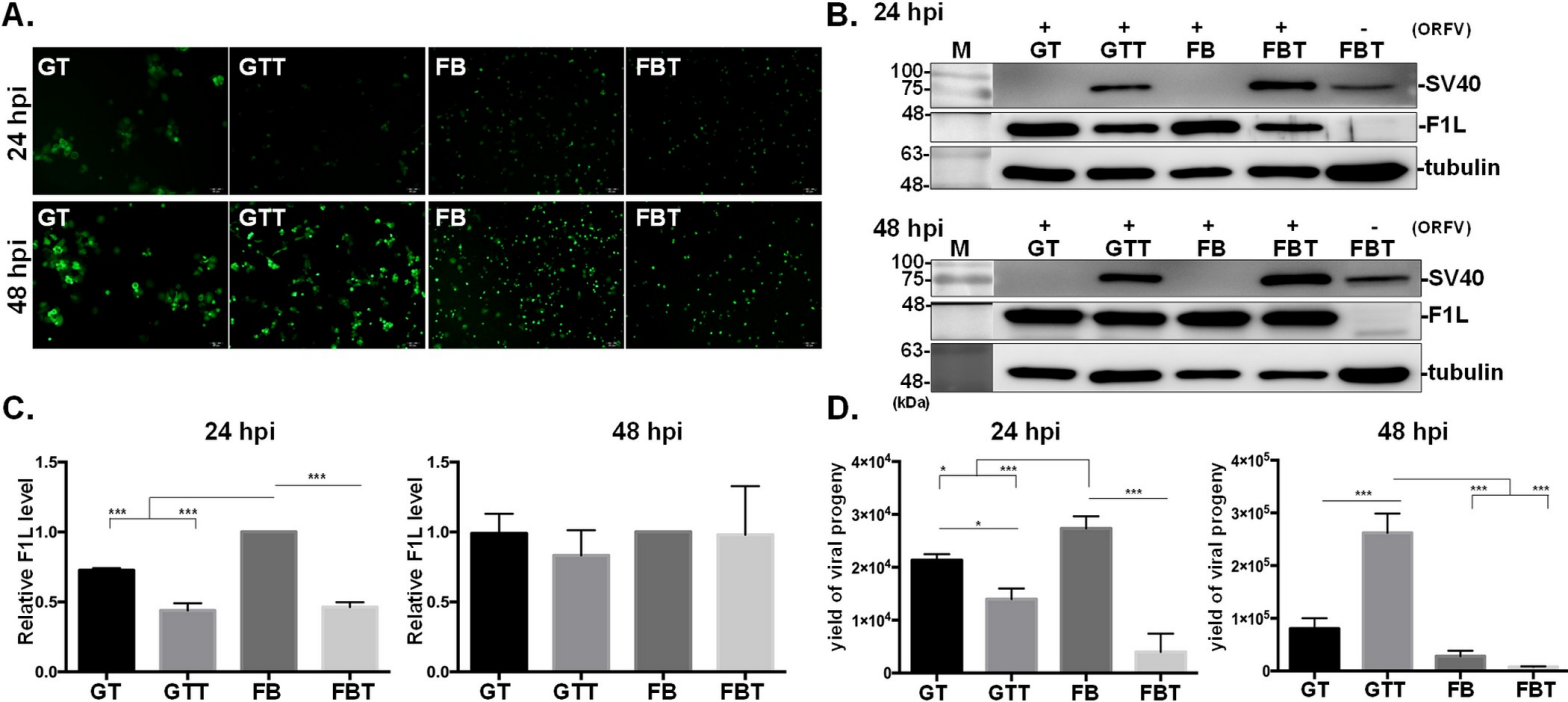
Susceptibility and the yield of viral progenies of ORFV in four different cell types. The infection of ORFV was initially evaluated by the fluorescence (A) and accumulated level of viral F1L protein (B and C). Cells were infected with OV20.0-GFP at 1 MOI and total cell lysate was harvested at 24 and 48 hpi for the detection of F1L expression (B, C) and viral yield (D). The F1L expression was firstly normalized with its respective α-tubulin level, and then the relative expression to primary FB cells was plotted (C). M, protein marker. The titers of viral progenies were determined by standard plaque assay on GTT cells and the relative yields of virus progenies were plotted (D). The student T-test was performed to evaluate the susceptibility of the primary cells and the yield of viral progenies in comparison with its respective SV40-transformed cells against ORFV. A p-value < 0.05 was considered significant and is represented by the asterisk sign, e.g. *, **, *** indicated p-value < 0.05, 0.01, 0.001, respectively.

Next, the susceptibility of SV40-transformed cells to ORFV was determined. Both goat primary cells and SV40-transformed cells were infected with OV20.0-GFP ORFV at 1 MOI for 24 and 48 hpi. As indicated in Fig 6A, all cells were permissive to ORFV infection as evidenced by the green fluorescence. Moreover, viral infection was further evaluated by the accumulation of the viral envelope protein, F1L. Among the four tested cells, FB cells had a significantly higher F1L level than the other three cell types at 24 hpi (Fig 6B and 6C). However, at 48 hpi there is no significant difference in the infection rates among the four tested cells as evidenced by the relative F1L level (Fig 6B and 6C).

Among the four cell types tested, FB cells yielded the highest viral progenies at 24 hpi while GTT yielded the highest viral progenies at 48 hpi (Fig 6D). Noticeably, the trend in F1L expression was in agreement with that in viral yield at 24 hpi (Fig 6D); the titer of viral progenies was highest in FB. Furthermore, with SV40 T antigen, the overall viral yield was lower than that in their corresponding parental cells at 24 hpi, whereas, the yield of viral progenies in GTT cells was higher than its primary cells at 48 hpi. Taken together, the immortalized cells with expression of SV40 T antigen are permissive to ORFV infection, although it may alter the susceptibility in a cell-type-specific manner.

## Discussion

Cell lines derived from various animal species have been used for propagation of ORFV, including monkey kidney Vero cells [49, 50], Madin-Darby ovine kidney cells [51] and Madin-Darby bovine kidney cells [52]. Previously, goat primary cells were generated for isolation of ORFV strain (Taipin) from field specimens and studies of viral pathogenesis and its interaction with the host cells [47, 53, 54]. However, the use of primary cells is restricted due to their limited proliferative capacity. To circumvent this disadvantage, in the present study, we transformed the previously established FB and GT primary cells by integrating the *SV40 T antigen* gene and subsequently characterized these transformed cells.

First of all, the susceptibility to ORFV infection was examined in several types of cells, including FB cells, GT cells, A549 cells, HEK 293T cells, Vero cells, and MDCK cells (Fig 1). The ORFV strain we used can replicate in the goat primary cells and the tested human cells. However, attenuated replication was observed in MDCK cells and surprisingly, our local strain of ORFV cannot infect Vero cells previously employed for ORFV propagation [48]. The data indicate that cell-tropism for ORFV infection may vary among viral strains. Moreover, in the tested cell types, FB and GT cells were the only cells that form plaques. Plaque forming ability is crucial for the isolation of viruses from fields and quantification of the infectious viral particles. Overall, the results demonstrated that goat primary cells derived from skin and testis were ideal for ORFV replication and isolation.

Viruses are capable of adapting to alternative hosts. For example, poxviruses acting as recombinant vaccine vector were obtained by serial passages in rabbit [55], mouse [56] or chicken derived cell lines [57]. Similarly, some ORFV strains were adapted to bovine [58, 59] or monkey cell lines [48, 60]. However, unexpected mutations might be introduced in the ORFV genome during adaption which may have impacts on its pathogenesis. For example, an attenuated ORFV strain D1701 lost the *E2L* gene while being passaged in a bovine kidney cell line [58, 59]. Very recently, ORFV strain variant D1701-V passaged in Vero cells lost *G2L* gene than its parental virus [48, 60], indicating that the use of cell lines originated from various hosts, and might lead to a different level of mutations in the genome of ORFV during isolation.

The DNA tumor virus, SV40, is known to induce immortalization (or transformation) in different cell origins, including rodent [61], sheep [35, 36, 38, 39], goats [29, 37, 39] and human [40–43] with frequencies as high as 100% in rodent [61] and dramatically lower (10^−8^ to 10^−5^) in human cells [62]. Among those, HEK293T is a commonly used SV40-transformed cell line. While this cell line could be permissive for ORFV infection, it is not an ideal host for plaque assay (Fig 1). It might be due to the restricted host range of ORFV.

Stable integration of SV40 T leads to changes in phenotypes and hence, an extension of the life span of the cells [61]. In our current study, FB and GT cells were isolated from ear skin or testis of a goat kid, respectively [11]. When these cells were immortalized by lentiviral transduction of SV40 T antigen, the morphology of transformed cells remained unaltered (Fig 2A). It has been suggested that the morphology change requires both large T and small t antigen. However, large T antigen alone can prolong the life span of the cells [61]. As compared with the life span of these two goat primary cells, GT cells can only be propagated up to 20 passages, which is slightly less than FB cells which can be propagated up to 30 passages, based on our own experience. However, with the expression of SV40 T antigen, FBT cells established in this study can be maintained at least up to 101 passages without compromising its susceptibility to ORFV (S2 Fig). A similar effect was also observed in GTT cells.

SV40-transformed cells were also characterized by their growth rates, susceptibility to ORFV and the ability to support viral replication. In general, higher FBS concentrations lead to faster cell growth. However, at a low FBS level (i.e. 2.5%), SV40-transformed FBT cells can proliferate and survive better than primary cells as evidenced by the steepness of the growth curve slope (Fig 4), suggesting that the growth of FBT cells was less dependent on FBS which is a typical characteristic of transformed cells [46].

Duncan and colleagues determined the susceptibility of normal and SV40-transformed human cells to reovirus infection and demonstrated that the yield of progeny virions in SV40- transformed cells was higher by two to three orders as compared to the normal human embryonic lung cell, WI-38 cells [63]. This may be related to the virus-mediated inhibition of cellular DNA synthesis in those cells [63]. In the present study, at 24 hpi, a significantly higher susceptibility of ORFV was shown in FB cells than FBT, based on F1L accumulation and the yield of virus progenies (Fig 6B, 6C and 6D), indicating that FB cells support viral propagation more effectively than the rest of tested cell types. However, at 48hpi, GTT produced the highest viral yield, while this trend was not in agreement with that of viral protein level; no significant difference in protein level among four cell types. Such a disagreement could be because that at the late stage of viral infection, cellular translation machinery has been shut down and cannot support further viral protein expression. Hence, the overall accumulation level of viral protein remains as plateau phase that may not be able to represent the difference in infection efficiency among four cell types analyzed. However, viral assembly (indicated as viral yield) is a late stage in viral replication cycle, which may not fully rely on cellular expression machinery, and therefore the influence by cell can be milder or delayed.

Although both of the primary cells were of goat origin, they have been isolated from different parts; FB was isolated from the ear while GT was isolated from the testis. Tropism specificity in cultured cells is controlled by specific receptors for virus binding and entry. However for poxviruses, no specific host-cell receptors were identified thus, the virus can probably attach and enter a wide range of mammalian cells, while its ability to fully complete the replication cycle varies significantly between cells of different lineages [20]. Unlike other poxviruses, infection of ORFV causes peripheral lesions and highly adapts to skin exclusively which might be one of the possible reasons for the increased viral progenies in FB cells than in GT cells. Moreover, it is known that viruses disseminate cell-free or cell-to-cell mode [64]. Although the spread of poxviruses mainly relies on cell-to-cell transmission, cell lysis also provides an alternative for cell-free transmission. Cell-to-cell mode of viral transmission greatly contributes to the viral pathogenesis of many viral infections as viruses will be delivered directly to their target cell receptors thus, increases binding and entry efficiency [64]. Furthermore, the viral spread of this transmission allows the virus to evade neutralizing antibodies which might be the case of increased ORFV infection in FB cells [65]. On the other hand, lower viral progenies with larger plaques of ORFV in GT cells could imply that ORFV infection is prone to cell lysis, which subsequently leads to a cell-free mode of spread [66].

Also, plaque size formed in FBT and GTT cells were comparably smaller than their respective primary cells (Fig 5). We suspected that SV40 T antigen expression may alter particular cellular signaling pathways which results not only in the discrepancy of cell growth rate, but also influenced viral replication. Firstly, FBT and GTT cells proliferate much faster than their parental cells leading to overgrowth of SV40-T-expressing cells during infection. In our observation, using the same amounts of viral inoculum, the higher cell density results in lower infection rates when tested in FBT and GTT cells. Hence, tight cell-cell junctions in FBT and GTT cells might reduce the infection rate of ORFV. Secondly, SV40-T antigen expression may inhibit apoptosis mediated by cellular proteins, such as p53, which possibly contributes to a slower progression of plaque formation in FBT and GTT cells. As demonstrated by other researches, SV40-T antigen binds to p53 and retinoblastoma protein, which are proposed to directly induce cell transformation [37–39]. Similarly, caprine or ovine cell lines immortalized with SV40-T antigen increased proliferation rate due to the inactivation of p53 and Rb [35, 37]. Inhibition of apoptosis may result in less cytolysis and then reduce the frequency of virus spread by suppressing the cell-free transmission route. Nevertheless, these assumptions are of importance and worthy of further investigation.

In sum, we have successfully generated immortalized goat fibroblast cells as well as immortalized goat testis cells suitable for isolation or propagation of ORFV. Despite the decrease in virus production and the reduction in the viral susceptibility of cells, the expression of SV40 T antigen signifies that the cells are still permissive to ORFV infection. Most importantly, these two types of transformed cells can be propagated in a substantial number of passages before senescence than their primary cells. However, cell signaling pathways mediated by the SV40-T antigen are worth further investigation to fully understand the mechanisms of immortalization of SV40 T antigen in goat primary cells and how this antigen contributes to ORFV infection.

## Supporting information

S1 Fig Morphology, genotype and growth curve of FB, FB-EV, FBT, GT, GT-EV and GTT. (A) Cell morphology was observed under bright-field microscopy. (B) PCR were used to further confirm the presence of the puromycin resistance gene (PuroR) and SV40 T antigen gene (T Ag) in goat cells. Two sets of cells, FB and FB-EV (C, left panel) and GT and GT-EV (C, right panel), were seeded at the same density (1x10^4^ cells/well) in a 48-well plate and were cultivated in DMEM supplemented with 10% FBS. Total cell counts were determined at 24 and 48 hours after seeded. The experiment was conducted in three repeats and the average cell counts were plotted.
(TIF)

S2 Fig Susceptibility of FBT (passage 101) to ORFV infection. Parental FB (FB-WT), and FBT (passage 101, P101) cells were infected with OV20.0-GFP at 1 MOI. After 24hpi, (A) infection rate was shown by the fluorescence protein (eGFP) in different passages of FBT and (B) western blot analysis of virus envelope protein (F1L) and T antigen. GAPDH served as an internal control. M: protein marker.
(TIF)

S1 Raw Data Original images for blots and gels. (PDF)
