# Supplementary figures and images for: Establishment and characterization of transformed goat primary cells by expression of simian virus 40 large T antigen for orf virus propagations
Source: PLoS One. 2019 Dec 5;14(12):e0226105. doi: 10.1371/journal.pone.0226105 (PMC6894772; doi:10.1371/journal.pone.0226105)

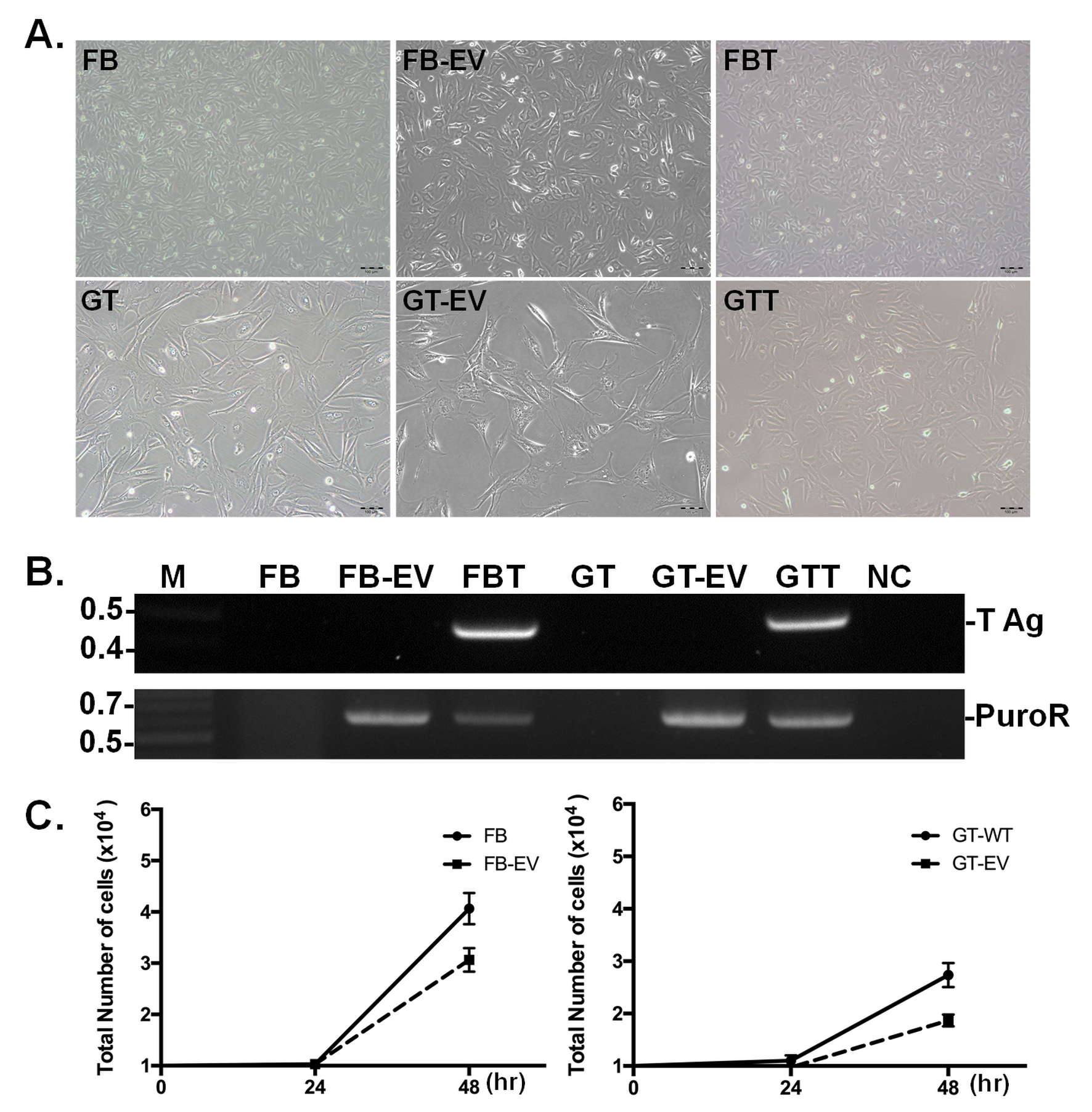

Supplement: S1 Fig — (A) Cell morphology was observed under bright-field microscopy. (B) PCR were used to further confirm the presence of the puromycin resistance gene (PuroR) and SV40 T antigen gene (T Ag) in goat cells. Two sets of cells, FB and FB-EV (C, left panel) and GT and GT-EV (C, right panel), were seeded at the same density (1x10^4^ cells/well) in a 48-well plate and were cultivated in DMEM supplemented with 10% FBS. Total cell counts were determined at 24 and 48 hours after seeded. The experiment was conducted in three repeats and the average cell counts were plotted. (TIF) [file pone.0226105.s001.tif]

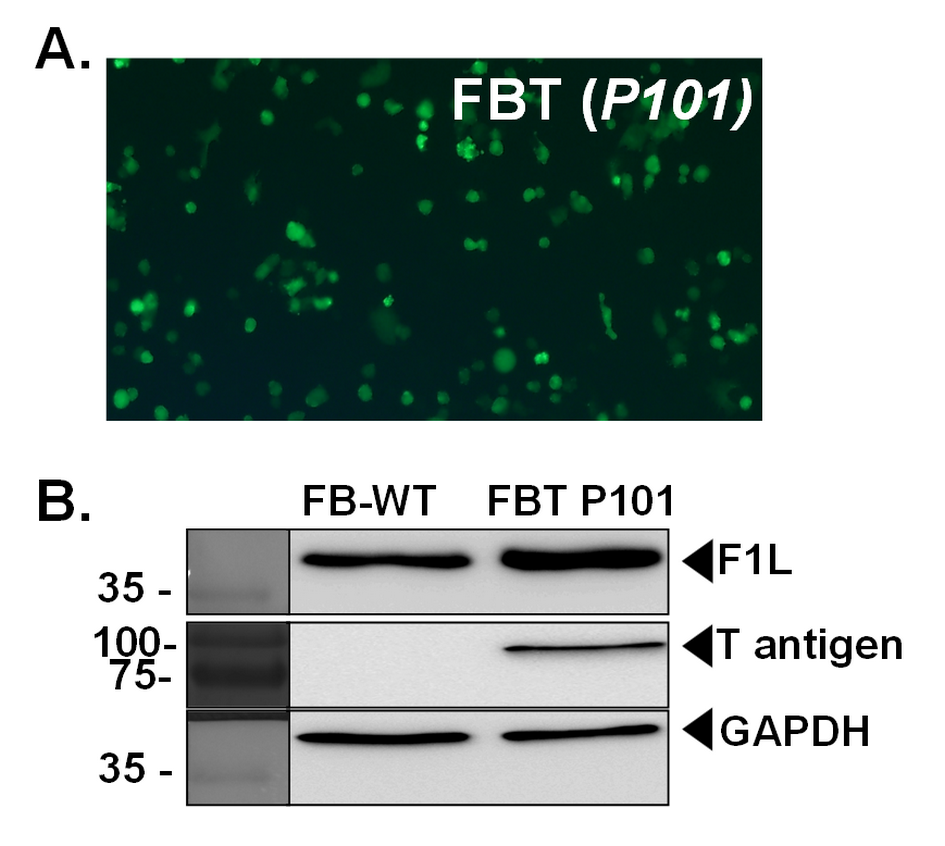

Supplement: S2 Fig — Parental FB (FB-WT), and FBT (passage 101, P101) cells were infected with OV20.0-GFP at 1 MOI. After 24hpi, (A) infection rate was shown by the fluorescence protein (eGFP) in different passages of FBT and (B) western blot analysis of virus envelope protein (F1L) and T antigen. GAPDH served as an internal control. M: protein marker. (TIF) [file pone.0226105.s002.tif]
