# Supplementary material for: Establishment and characterization of transformed goat primary cells by expression of simian virus 40 large T antigen for orf virus propagations
Source: PLoS One. 2019 Dec 5;14(12):e0226105. doi: 10.1371/journal.pone.0226105 (PMC6894772; doi:10.1371/journal.pone.0226105)
Supplement: S1 Raw Data — (PDF) [file pone.0226105.s003.pdf]

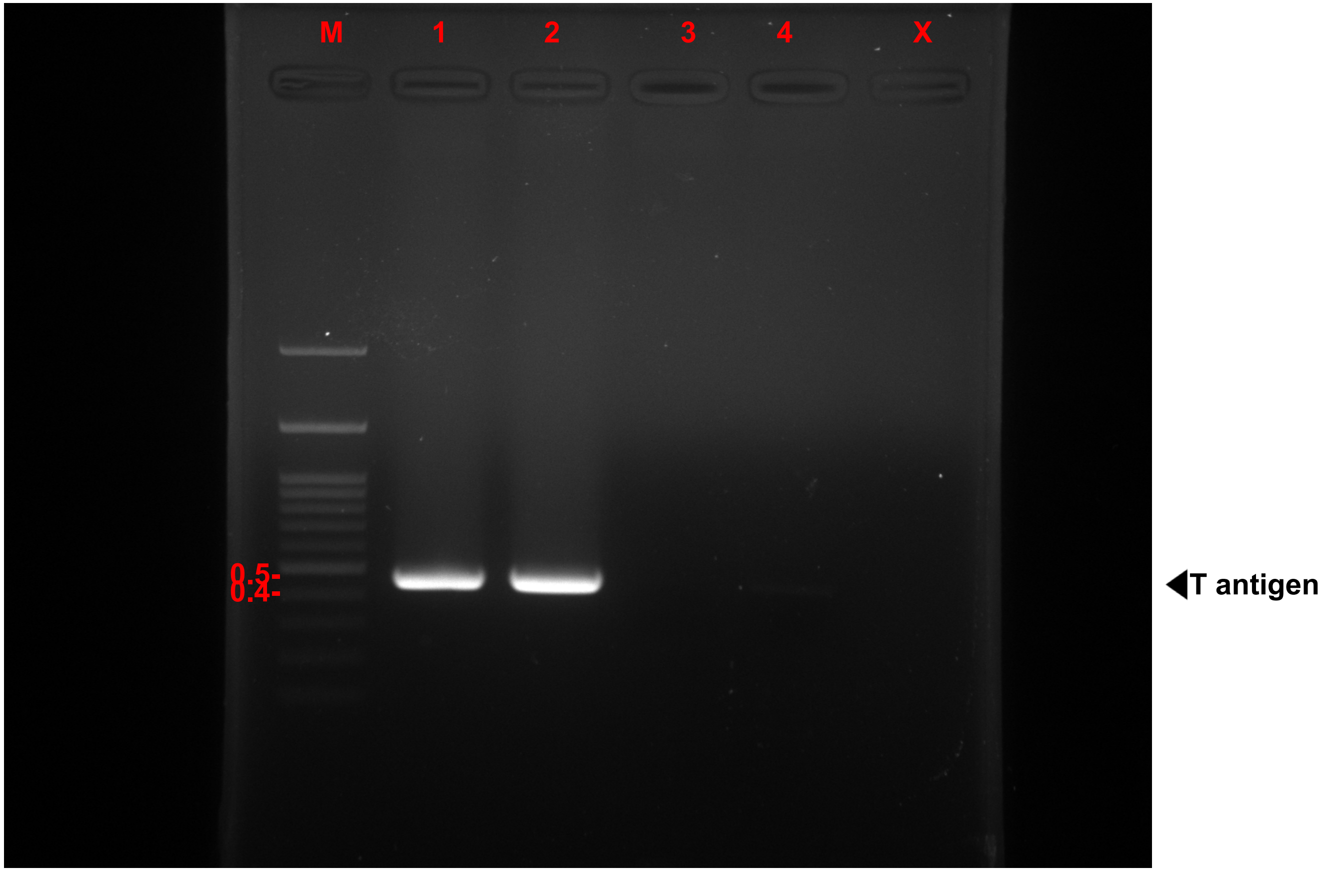

Original Image of Fig 2B.
Fig 2B image was produced by cropping the image above as marker for lane M; lanes 1 and 2 for GTT; lane 3 for GT and lane 4 as negative control (NC). Image was taken using Quantum ST4 under automatic exposure of 0.2 seconds.

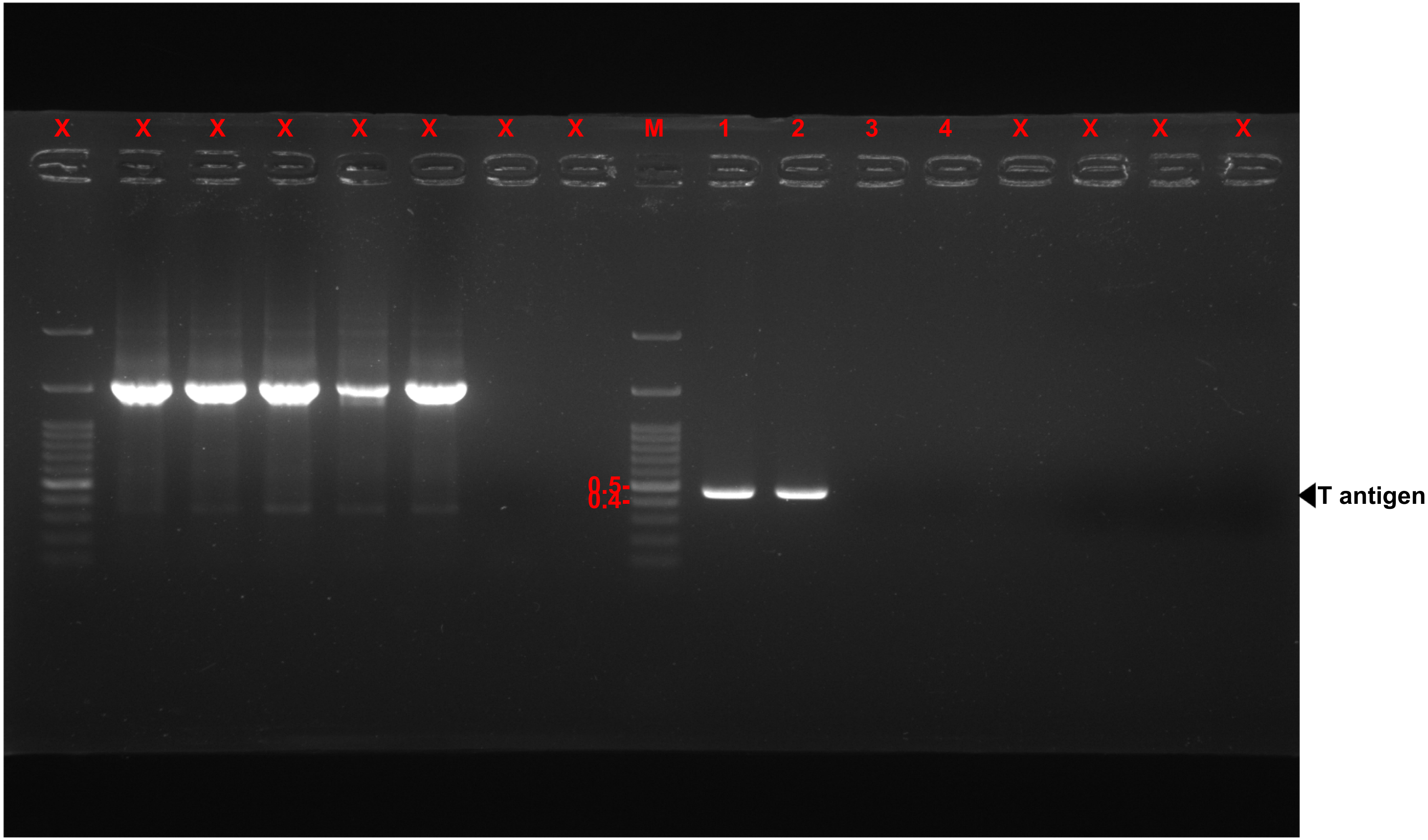

Original Image of Fig 2B.
Fig 2B image was produced by cropping the image above as marker for lane M; lanes 1 and 2 for FBT; lane 3 for FB and lane 4 as negative control (NC). Image was taken using Quantum ST4 under automatic exposure of 0.2 seconds.

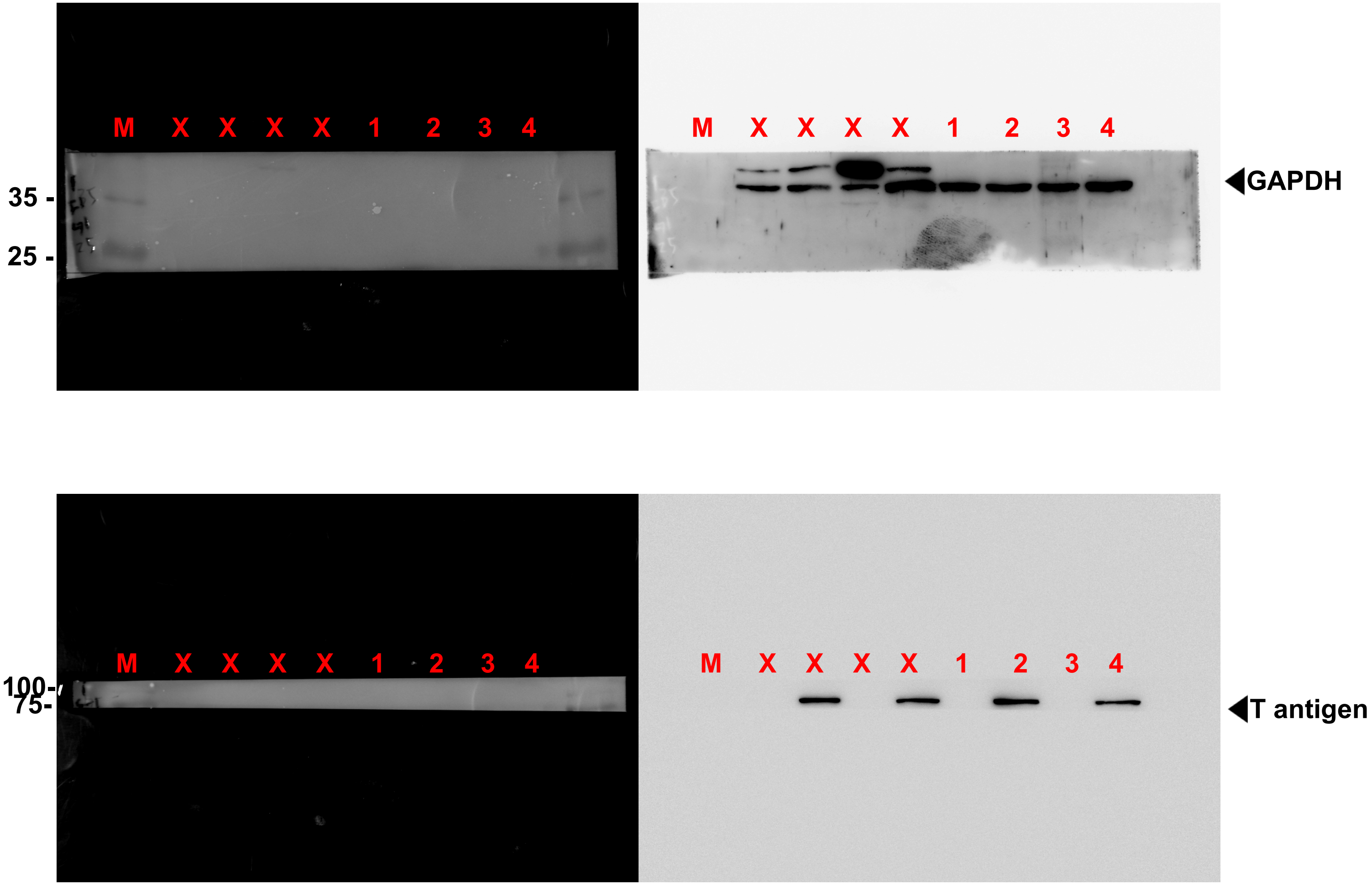

Original image used in Fig 2C.
Fig 2C was produced from these two blots. 10 uL of protein samples were loaded into each well. Gels were ran and transferred together under the same western blot conditions. Each blot was incubated with the desired primary antibody followed by their respective secondary antibodies as described in the materials and methods. Images on the left, are photos taken under digitalization while on the right, are photos taken using chemiluminescence. All photos were taken using the same exposure time, 1/60s for digitalization and 40 seconds for chemiluminescence. "X" in the figure above are results which are not part of the final figure. M, marker; 1, GT; 2, GTT; 3, FB; 4, FBT.

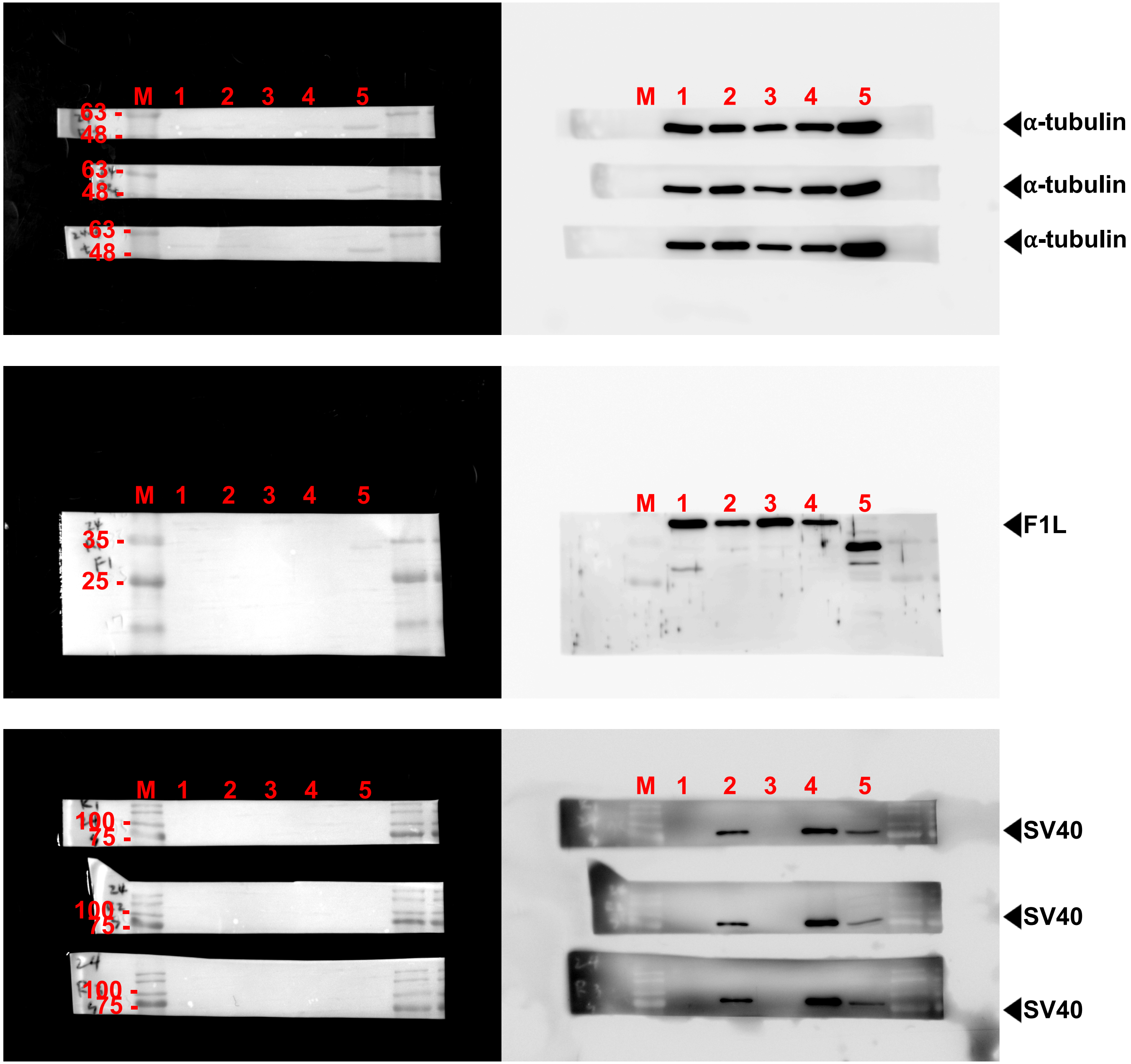

Original image used in Fig 6B (24hpi).

Fig 6B (24hpi) was produced from these blots. 10 uL of protein samples were loaded into each well. Gels were ran and transferred together under the same western blot conditions. Each blot was incubated with the desired primary antibody followed by their respective secondary antibodies as described in the materials and methods. Images on the left, are photos taken under digitalization while on the right, are photos taken using chemiluminescence. All photos were taken using the same exposure time, 1/100s for digitalization and 40 seconds for chemiluminescence. ”X” in the figure above are results which are not part of the final figure. M, marker; 1, GT (infected); 2, GTT(infected); 3, FB (infected); 4, FBT (infected); 5, FBT (mock).

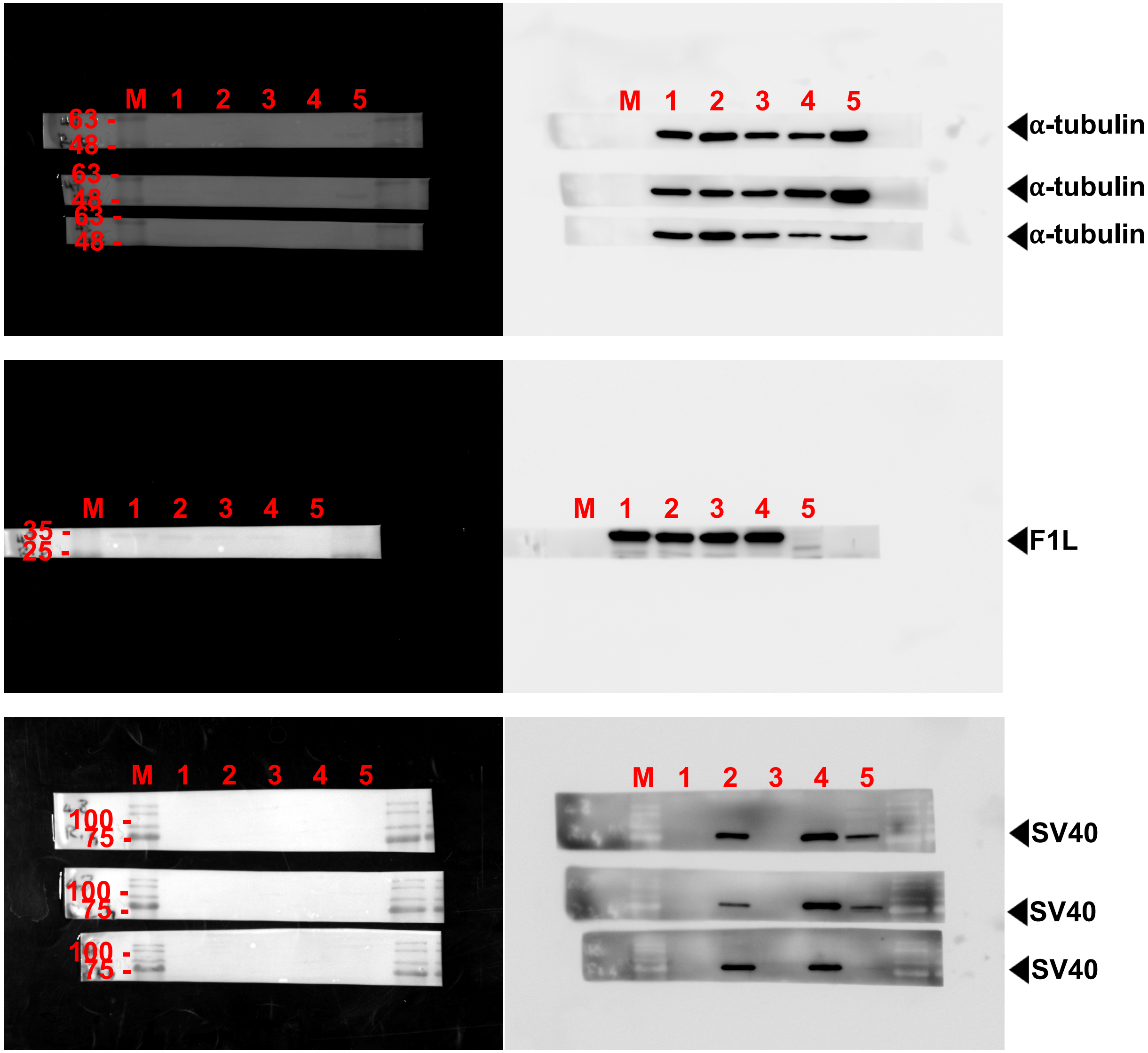

Original image used in Fig 6B (48hpi).

Fig 6B (48hpi) was produced from these blots. 10 uL of protein samples were loaded into each well. Gels were ran and transferred together under the same western blot conditions. Each blot was incubated with the desired primary antibody followed by their respective secondary antibodies as described in the materials and methods. Images on the left, are photos taken under digitalization while on the right, are photos taken using chemiluminescence. All photos were taken using the same exposure time, 1/100s for digitalization and 40 seconds for chemiluminescence. ”X” in the figure above are results which are not part of the final figure. M, marker; 1, GT (infected); 2, GTT(infected); 3, FB (infected); 4, FBT (infected); 5, FBT (mock).

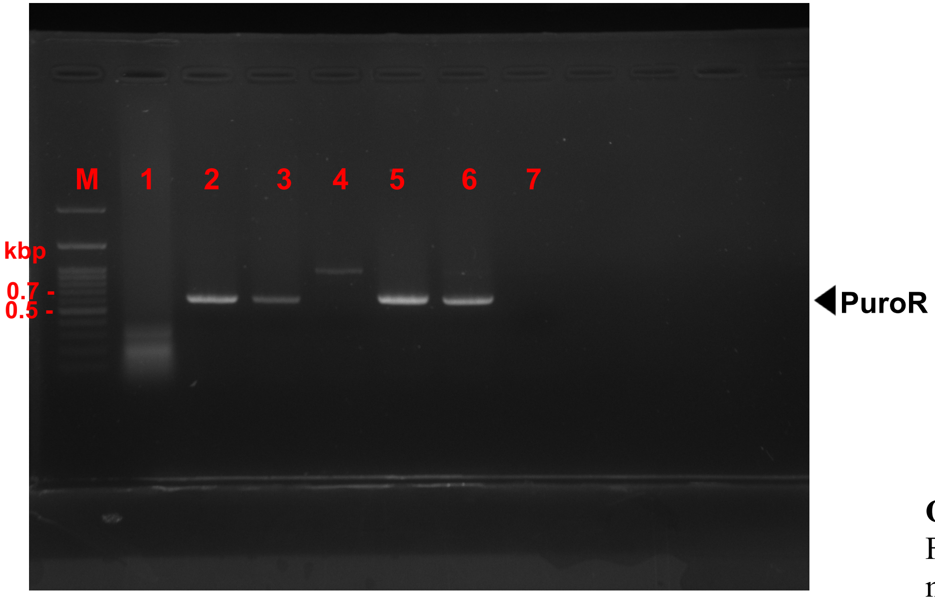

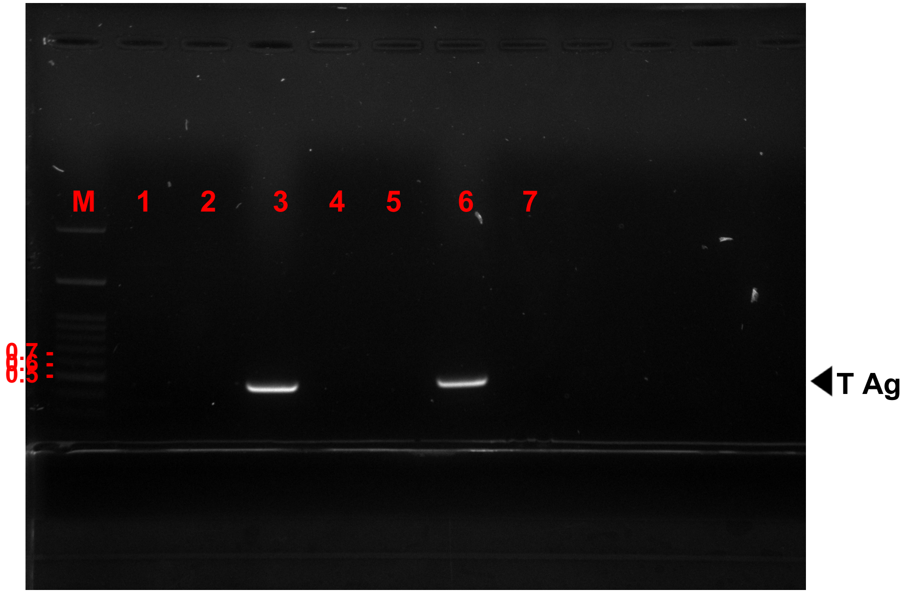

Original Image of Fig S1B.
Fig S1B image was produced by cropping the image above as marker for lane M; lane 1 for FB; lane 2 for FB-EV ; lane 3 for FBT; lane 4 for GT; lane 5 for GT-EV; lane 6 for GTT and lane 7 for negative control (NC). Image was taken using Quantum ST4 under automatic exposure of 0.2 seconds.

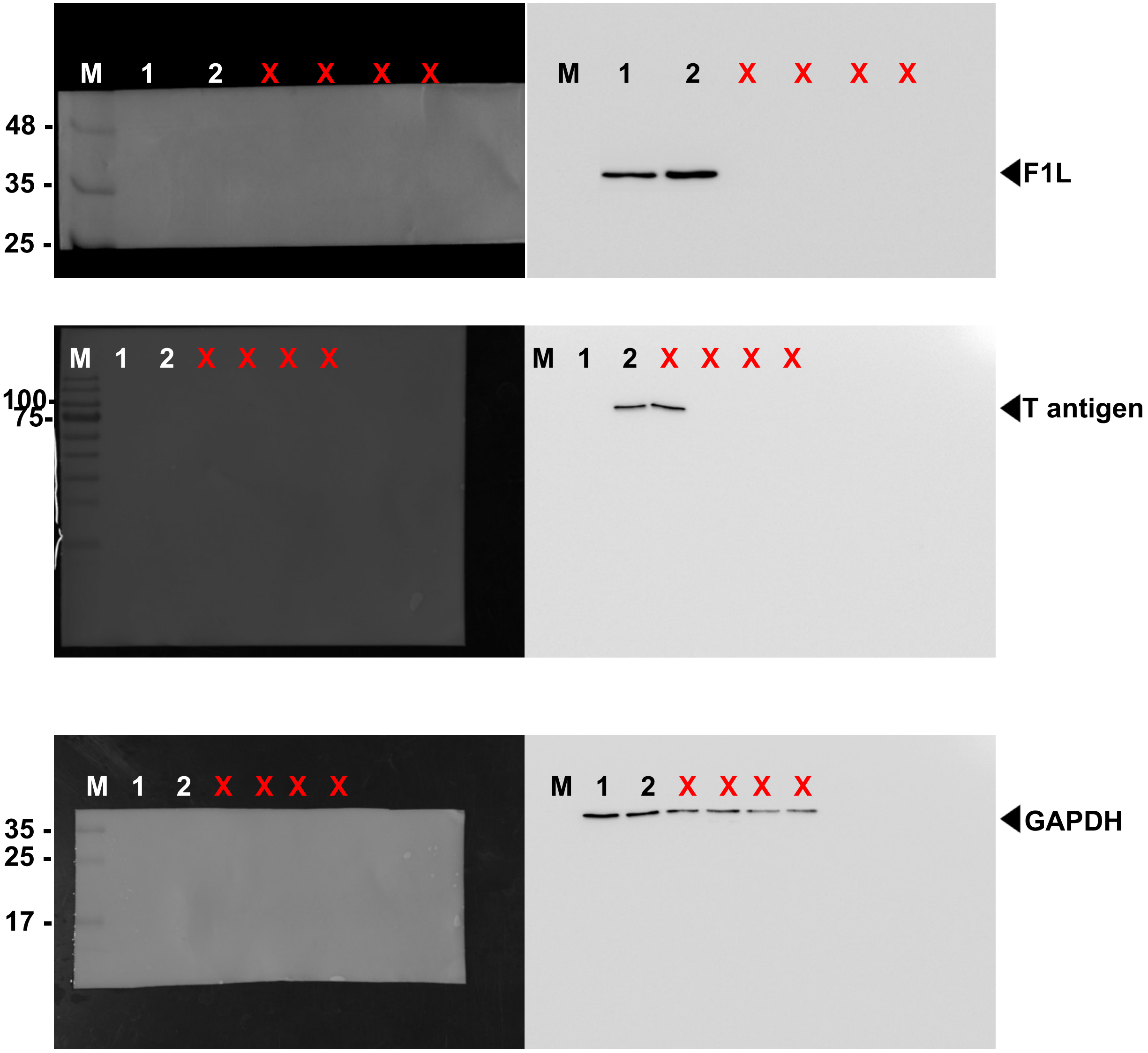

Original image used in figure S2B. Figure S1B was produced from these three blots. 10 uL of protein samples were loaded into each well. Gels were ran and transferred together under the same western blot conditions. Each blot was incubated with the desired primary antibody followed by their respective secondary antibodies as described in the materials and methods. Images on the left, are photos taken under digitalization while on the right, are photos taken using chemiluminescence. All photos were taken using the same exposure time, 1/60s for digitalization and 30 seconds for chemiluminescence. ”X” in the figure above are results which are not part of the final figure. M, marker; 1, FB-WT; 2, FBT P101.
